# Transport Vesicle Tethering at the Trans Golgi Network: Coiled Coil Proteins in Action

**DOI:** 10.3389/fcell.2016.00018

**Published:** 2016-03-15

**Authors:** Pak-yan P. Cheung, Suzanne R. Pfeffer

**Affiliations:** Department of Biochemistry, Stanford University School of MedicineStanford, CA, USA

**Keywords:** coiled coil protein, Golgi, membrane traffic, transport vesicle, atomic force microscopy

## Abstract

The Golgi complex is decorated with so-called Golgin proteins that share a common feature: a large proportion of their amino acid sequences are predicted to form coiled-coil structures. The possible presence of extensive coiled coils implies that these proteins are highly elongated molecules that can extend a significant distance from the Golgi surface. This property would help them to capture or trap inbound transport vesicles and to tether Golgi mini-stacks together. This review will summarize our current understanding of coiled coil tethers that are needed for the receipt of transport vesicles at the trans Golgi network (TGN). How do long tethering proteins actually catch vesicles? Golgi-associated, coiled coil tethers contain numerous binding sites for small GTPases, SNARE proteins, and vesicle coat proteins. How are these interactions coordinated and are any or all of them important for the tethering process? Progress toward understanding these questions and remaining, unresolved mysteries will be discussed.

Membrane trafficking involves the collection of cargo into transport vesicles, translocation of vesicles along cytoskeletal tracks, and tethering, docking, and fusion of vesicles at their target membranes. Tethering involves the initial capture of transport vesicle at a distance from the target membrane and the process by which the two membranes are brought together into close proximity. Tethering appears to be an important means to both enhance the efficiency and control the accuracy of subsequent, SNARE-mediated membrane fusion events (Pfeffer, 1999).

At least 15 different, long, coiled-coil-containing proteins decorate the Golgi complex and have been nicknamed, Golgins (Short et al., 2005; Munro, 2011). Given that the rise per residue in a coiled coil is typically 0.148 nm, the majority of Golgins could theoretically extend more than 100 nm in length. This feature would make them well suited to connect membranes at a distance. In this review, we will discuss how a subset of long coiled coil proteins may function as tethers at the trans Golgi network (TGN) and cooperate with their binding partners to both localize to specific membrane compartments and catch inbound vesicles.

## TGN golgins

**Figure 1:**
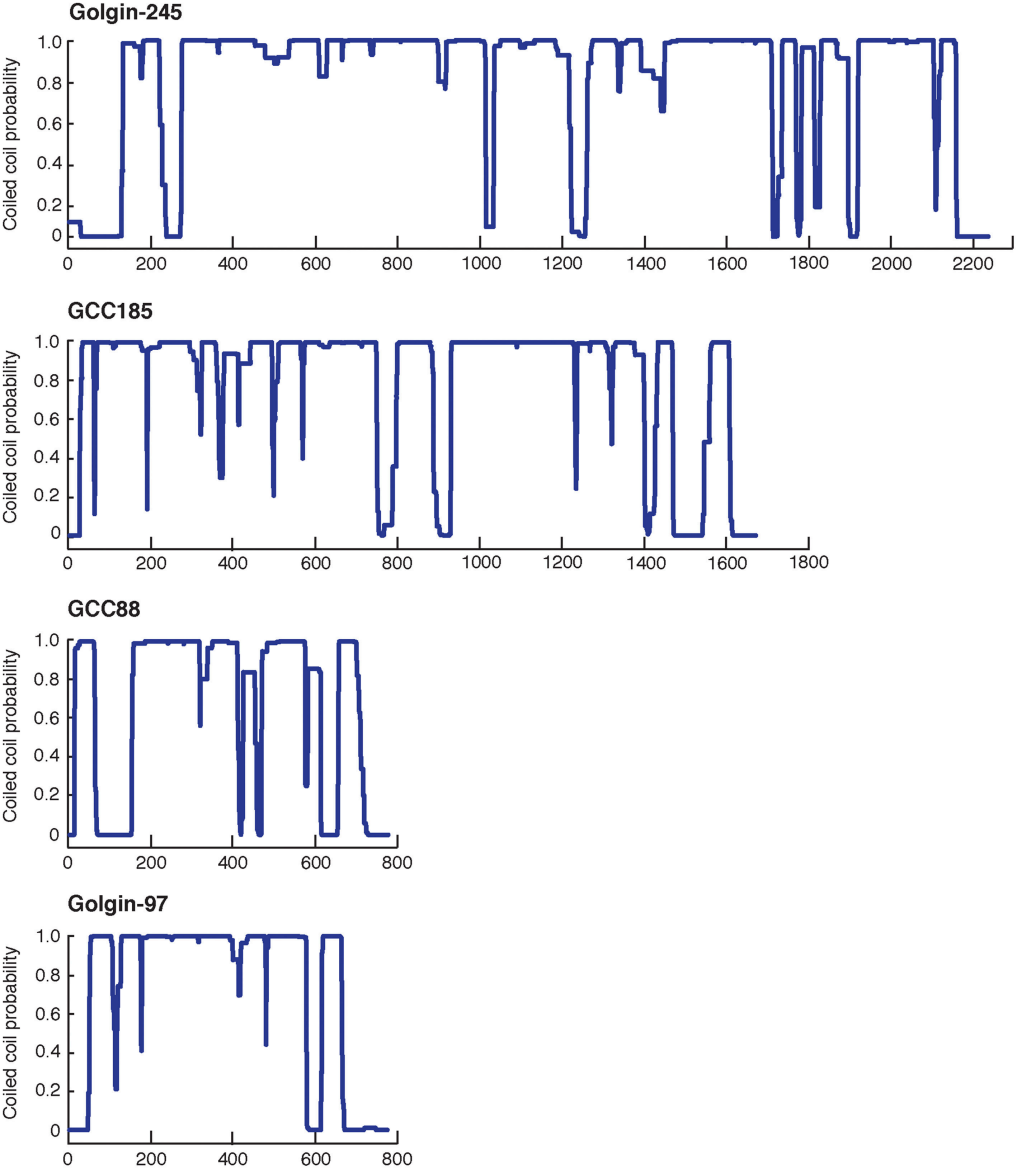
**Predicted probability of each amino acid in the sequences of the four TGN Golgins to form a coiled-coil structure**. Top to bottom: Golgin-245, GCC185, GCC88, and Golgin-97. The central coiled coil region and the adjacent breaks of GCC185 and Golgin-245 likely form a central bubble.

At the TGN in human cells, the four Golgins, Golgin-245, Golgin-97, GCC185, and GCC88, are localized to at least three distinct subdomains (Luke et al., 2003; Derby et al., 2004; Gleeson et al., 2004; Brown et al., 2011). [Because Golgin refers to a person, we prefer to refer to these proteins using an upper case: **G**olgin.] These dimeric Golgin proteins are present both in the cytosol and on membranes (about 50:50), and require at least one small GTPase for their TGN recruitment. Figure 1 presents their predicted content of coiled coil structure; note the significant sequence length differences, and the clear breaks in the predicted coiled coils, about halfway along the sequence of the two longer proteins. As described below, the N-terminus of purified GCC185 is splayed and Y-shaped (Cheung et al., 2015), a feature shared with the cis-Golgi localized, GM130 protein (Ishida et al., 2015), and likely also, with the other trans Golgi-localized Golgins shown here (because their N-termini show a lower probability of coiled-coil dimerization). The less well characterized Golgin, TMF, also has no predicted coiled coil in the first ~400 amino acids of its 1093 residue protein sequence, but significant coiled coil probability thereafter (not shown).

**Figure 2:**
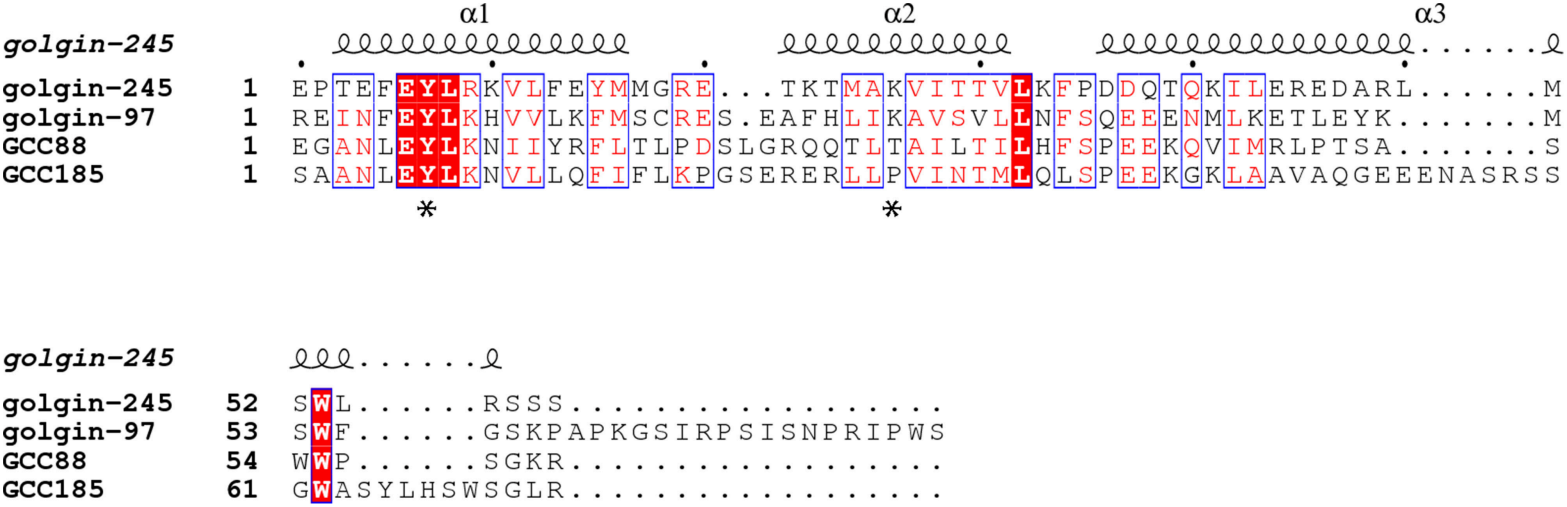
**Sequence alignment of the GRIP domains of human Golgin-245, Golgin-97, GCC88, and GCC185 based on the crystal structure of Golgin-245 generated from PROMALS3D (Pei et al., 2008) and ESPript 3.0 (Robert and Gouet, 2014)**. The secondary structures of human Golgin-245 are shown above. Invariant residues are in white with red background; similar residues (with global scores between 0.7 and 1.0) are in red, framed in blue, and other residues are in black. Asterisks indicate the invariant tyrosine that is critical for Golgin-245 and Golgin-97 localization and Arl1 binding, as well as the proline in GCC185 that may break helix 2.

Golgin-245 and Golgin-97, originally identified as Golgi-localized autoantigens (Fritzler et al., 1995; Erlich et al., 1996; Griffith et al., 1997), were later found to belong to a family of proteins containing a conserved, 50-60 amino acid, C-terminal sequence called a GRIP domain (Kjer-Nielsen et al., 1999; Munro and Nichols, 1999). The GRIP domain comprises a binding site for the small GTPase, Arl1, and is important for Golgi targeting of these two Golgins (Figure 2). A conserved GRIP domain tyrosine (Y2177 in Golgin-245; Figure 2 asterisk) is essential for both Golgi targeting and Arl1 interaction (Barr, 1999; Kjer-Nielsen et al., 1999; Munro and Nichols, 1999; Lu and Hong, 2003).

Golgin-245's GRIP domain is a homodimer that interacts with two molecules of GTP-bound Arl1. The GRIP domain is comprised of three antiparallel α helices arranged in an S-shaped configuration (Panic et al., 2003; Wu et al., 2004); the Arl1:GRIP domain complex is anchored to the membrane via Arl1's N-terminal myristoyl group, and possibly also, a GRIP domain tryptophan residue (Panic et al., 2003; Wu et al., 2004; Lu et al., 2006). In yeast, TGN recruitment of the GRIP domain-containing, Imh1 protein is regulated by Arl3p and the small transmembrane protein, Sys1 (Gangi Setty et al., 2003; Behnia et al., 2004).

Consistent with the importance of Arl1 binding for their TGN localizations, Golgin-245 and Golgin-97 re-localized to early endosomes when Arl1 was artificially targeted to that compartment (Derby et al., 2004). In contrast, GCC185 and GCC88 interact weakly with Arl1 (van Valkenburgh et al., 2001; Lu and Hong, 2003; Derby et al., 2004; Reddy et al., 2006) and were not relocalized under these conditions. *Drosophila* GCC88 but not GCC185, showed dependence on Arl1 for Golgi localization (Torres et al., 2014). Indeed, mutation of GCC185's conserved GRIP domain tyrosine had only a minimal effect on its localization (Barr, 1999; Kjer-Nielsen et al., 1999; Munro and Nichols, 1999; Lu and Hong, 2003; Derby et al., 2004; Reddy et al., 2006; Burguete et al., 2008), suggesting that another mechanism is likely in play. As described below, Rab6A facilitates Arl1 binding to GCC185's GRIP domain *in vitro* (Burguete et al., 2008), and can override the loss of the key tyrosine to permit Arl1 binding. In this manner, two small GTPases may cooperate to anchor GCC185 at the TGN.

Alignment of the four, human GRIP domain sequences also provides clues to their differences in Arl1 interaction (Figure 2). A lysine residue (K2196) located in the middle of Golgin-245's second GRIP domain alpha helix (Figure 2 asterisk) forms a hydrogen bond with an Arl1 serine residue at the binding interface (Panic et al., 2003; Wu et al., 2004). In GCC185, the equivalent residue is instead, a proline, which very likely alters the structure of the second GRIP domain alpha helix, and may explain the difference in Arl1 binding between GRIP domains. In human GCC88, a threonine replaces the lysine and may influence Arl1 binding to a lesser extent.

## Rab GTPase-mediated tether recruitment

At least 15 different Rab GTPases may be present on the Golgi, and several interact with the TGN Golgins; Rab6A is particularly important for Golgi tether recruitment (Table 1). Rab6 recruits the p150^glued^ subunit of the dynactin complex and the TGN-localized, coiled coil protein, bicaudal D (Short et al., 2002). Rab6 interacts with bicaudal D's C-terminal coiled coil (Matanis et al., 2002); coordination between bicaudal D1/D2, Rab6 and the dynein-dynactin complex targets bicaudal D1/D2 to the Golgi (Short et al., 2002). The yeast protein, Sgm1 is recruited to the Golgi by interacting with the Rab6 ortholog, Ypt6 (Siniossoglou and Pelham, 2001). Sgm1's mammalian homolog, TMF, also interacts with three mammalian orthologs of Ypt6: Rab6A, Rab6A', and Rab6B, via the C-terminal coiled coil domain, for Golgi localization (Fridmann-Sirkis et al., 2004; Siniossoglou, 2005; Yamane et al., 2007). Moreover, as described below, the kinesin-family motor, KIF1C can act as a tether under certain circumstances and is recruited to the Golgi via C-terminal, Rab6A binding (Lee et al., 2015).

**Table 1:** **TGN Golgins and tether-like proteins and binding partners**.

**Protein**	**Small GTPases**	**SNAREs, coat proteins**	**Others**
GCC88	Arl1, Rab6, 19, 30, Panic et al., 2003; Sinka et al., 2008		
	Rab2, 30, Gillingham et al., 2014		
GCC185	Rab9A, Reddy et al., 2006	STX16, Ganley et al., 2008	CLASP2, Efimov et al., 2007
	Arl1, Panic et al., 2003; Burguete et al., 2008	AP-1, Brown et al., 2011	CLASP1α
	Arl4A, Lin et al., 2011		CLASP2γ, Brown et al., 2011
	Rab6A, Burguete et al., 2008		CLASP binding may require Arl4, Lin et al., 2011
	Rab1A,1B, 2A, 2B, 6A, 6B, 9A, 9B, 15, 27B, 30, 33B, 35, 36, Sinka et al., 2008; Hayes et al., 2009; Gillingham et al., 2014		
Golgin97	Rab6, Barr, 1999		FIP1/RCP (Rab11-binding protein), Jing et al., 2010
	Arl1, Panic et al., 2003		
	Rab6, 19, 30, Sinka et al., 2008; Gillingham et al., 2014		
Golgin245	Rab6, Barr, 1999		MACF1, Kakinuma et al., 2004
	Arl1, Arl3, van Valkenburgh et al., 2001; Panic et al., 2003		
	Rab2, 30, Sinka et al., 2008; Gillingham et al., 2014		
Bicaudal D	Rab6, Short et al., 2002; Matanis et al., 2002		Dynactin, Dynein, Hoogenraad et al., 2001; Matanis et al., 2002; Short et al., 2002
	Rab30; Rab2, 6, 30, 39, Sinka et al., 2008; Gillingham et al., 2014		
KIF1C	Rab6A, Lee et al., 2015		PTPD1, Dorner et al., 1998
			Bicaudal D-related 1, Schlager et al., 2010
			14-3-3 proteins, Dorner et al., 1999

Rab6A binds GCC185 just upstream of the C-terminal GRIP domain, which facilitates Arl1 binding to this GRIP domain *in vitro* (Burguete et al., 2008). A stable attachment platform supporting GCC185 on the TGN is formed by anchoring two Rab6 molecules to the TGN membrane with the four C-terminal geranylgeranyl groups accommodated by an extended, Rab GTPase hypervariable domain; insertion of Arl1 myristoyl groups into the membrane (Panic et al., 2003; Wu et al., 2004) and possibly the membrane insertion of GCC185's C-terminus (Panic et al., 2003) would further stabilize this interaction. This model explains the inability of Arl1 alone to mediate GCC185's Golgi localization.

Houghton et al. (2009) challenged the roles of Arl1 and Rab6 in GCC185 localization. In that study, those workers used siRNA to achieve 80% depletion of Rab6; under those conditions, they failed to alter the Golgi association of GCC185. Burguete et al. (2008) required at least 90% depletion of Rab6 to alter GCC185 localization, which readily explains this discrepancy. GCC185 also binds CLASP proteins and other Rab GTPases, all of which may contribute to the Golgi localization of this Golgin. Nevertheless, the only fragment of GCC185 that is sufficient for Golgi localization is the C-terminal region comprised of the GRIP and Rab6 binding domains (Burguete et al., 2008; Hayes et al., 2009). Given the overall, structural conservation of GRIP domain Golgins, it seems unlikely that GCC185 would be very functionally distinct from other TGN-localized, GRIP domain proteins, despite the fact that its Golgi localization mechanism may be somewhat more complex. Moreover, recent work (Bardin et al., 2015) supports a model in which Rab6 may cooperate with Arl1 (and perhaps additional factors) for mammalian GCC185 Golgi localization. Note that targeting of Drosophila GCC185 to the TGN is not affected by the absence of either or both Arl1 and Rab6 (Torres et al., 2014), but the C-terminal half of the Drosophila protein (residues 582–1127) is only 26% identical to the corresponding region of the 1684 residue human GCC185 protein (46% similar with major gaps).

## How do tethers catch vesicles?

Little is known about how long, coiled coil Golgins actually capture transport vesicles. Their roles as tethers have been inferred by the phenotypes seen upon their depletion from cultured cells. Golgi fragmentation is always detected; in some cases, transport vesicle intermediates also accumulate in the cytoplasm. Thus, long tethers can have dual roles, and work on GCC185 has shown that Golgi ribbon structure stabilization and transport vesicle tethering can utilize different regions of this protein (Brown et al., 2011).

By definition, tethering proteins should have the capacity to bind transport vesicles directly, to link them to the corresponding target membranes. Yet to date, few TGN tethers have been clearly shown to do this. Reflecting tethering events that take place earlier in the Golgi, Warren and colleagues used purified CASP and p115 proteins to capture distinct classes of transport vesicles enriched in Golgin-84 or p24 proteins (Malsam et al., 2005). However, the precise domains of CASP and p115 responsible for vesicle binding is still not known, and how or whether the proteins bring these vesicles closer to their corresponding target membranes is also unknown.

The notion that tethering proteins use their N-termini to “catch” vesicles, while being anchored at target membranes via their C-termini, was reinforced upon characterization of GMAP-210. This cis-Golgi tether has a curvature-sensing, amphipathic lipid-packing sensor (ALPS) motif at its extreme N-terminus that binds liposomes of high membrane curvature (radius < 50 nm) but not flat membranes *in vitro* (Drin et al., 2007, 2008). Upon heterologous expression in yeast cells (that normally lack GMAP-210), this ALPS motif was targeted to uncoated vesicles carrying markers from the ER-Golgi interface and late Golgi (Pranke et al., 2011), which likely reflects their small size and/or specific lipid compositions. In mammalian cells, GMAP-210's N-terminal ALPS domain was recently shown to be sufficient to tether vesicles upon relocation to mitochondria (Sato et al., 2015; see also Wong and Munro, 2014). Such relocation did not require Rab2 binding sites located in the central coiled coil region of GMAP-210; these sites were, however, essential for GMAP-210's ability to maintain normal Golgi structure. From this, Lowe and colleagues concluded that Rab2's role is downstream of N-terminal, ALPS motif-mediated vesicle binding (Sato et al., 2015), and again separable from vesicle capture.

GMAP-210 is the only Golgin that contains an ALPS motif, thus other Golgins must tether vesicles by another mechanism. Several Golgi tethers interact with vesicle coat proteins (Cai et al., 2007; Bröcker et al., 2010). For example, cis-Golgi localized p115 can interact directly with βCOP to facilitate vesicle tethering (Guo et al., 2008). The interaction maps to p115's N-terminal, globular domain and βCOP's so-called FW motif. Thus, p115 uses its N-terminal heads to bind the vesicle coat and its coiled coil domain to bind to Rab1 (Beard et al., 2005), syntaxin-5, and GOS-28 on target membranes (Shorter et al., 2002; Guo et al., 2008).

GCC185 can bind the AP-1 clathrin adaptor that likely decorates the vesicles it captures (Brown et al., 2011; Table 1). AP-1 interacts with the cytoplasmic domains of mannose 6-phosphate receptors [(MPRs); Glickman et al., 1989] and functions in the both the export of MPRs from the TGN to endosomes as well as their transport from late endosomes back to the TGN (Meyer et al., 2000; Braulke and Bonifacino, 2009). GCC185's AP-1 binding site near the middle of this tether (just beyond the central bubble, see below), is required for the overall process of MPR-vesicle tethering (Brown et al., 2011).

**Figure 3:**
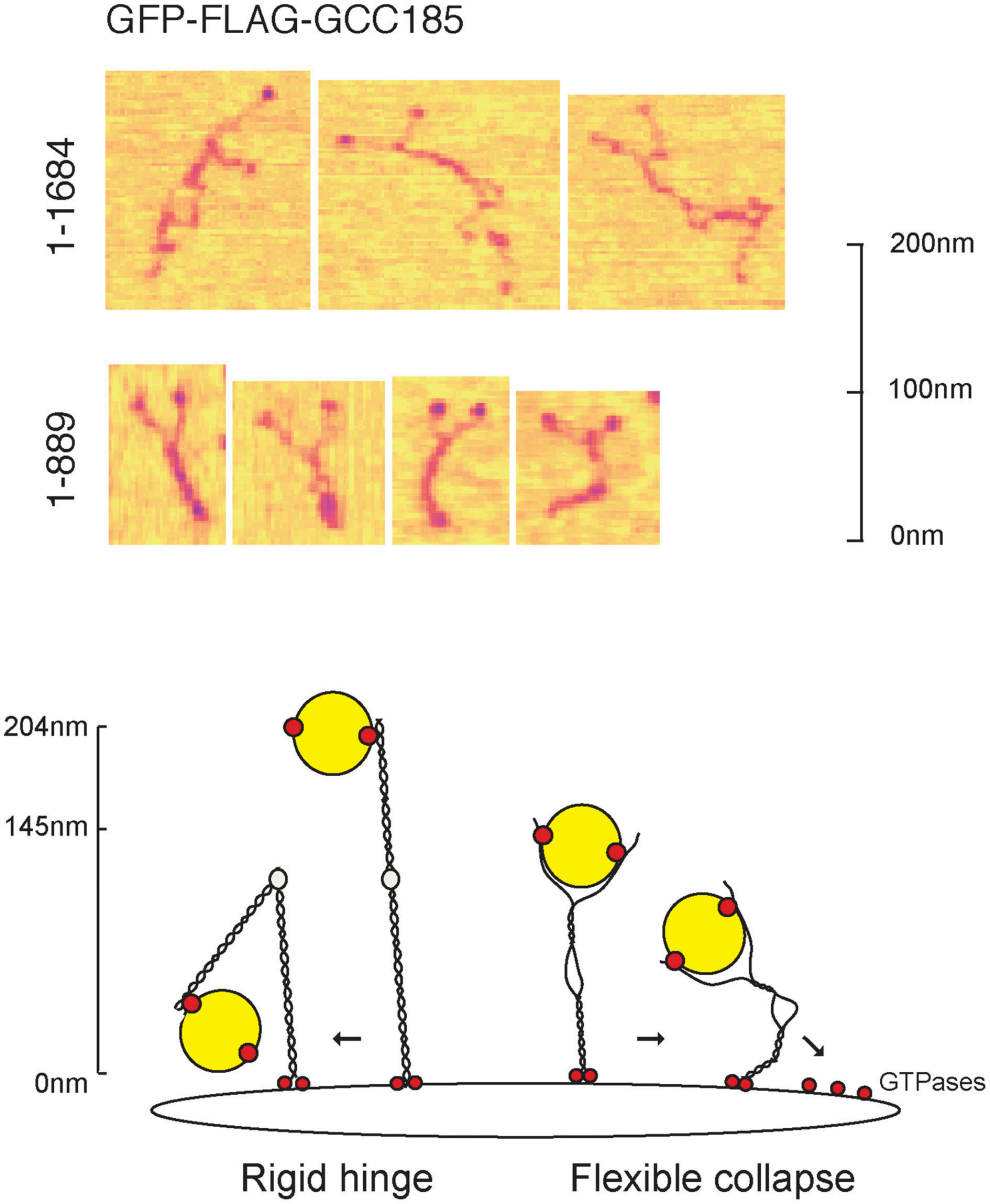
**Top**, the splayed and flexible structure of full length GCC185 (residues 1-1684) detected by atomic force microscopy (AFM). Purified, full length, GFP-GCC185 and purified, N-terminal half-molecules (residues 1-889) deposited on mica (from Cheung et al., 2015). Readily discerned are the N-terminal GFP spheres; most of the molecules showed splayed N-termini, followed by a dimeric region, an unwound central bubble, followed by a short coil and C-terminal GRIP domains. The central bubble sequences were essential for function and could be replaced by random coils, demonstrating that this region needs only to be flexible to sustain vesicle docking at the Golgi. **Bottom**, collapse model for vesicle tethering at the TGN. Previous models, based upon GMAP210, have suggested that rigid tethers may bend in the middle to bring vesicles closer to the membrane. The AFM studies (Cheung et al., 2015) suggest that tethers may be much more flexible than previously thought, and may collapse onto the membrane surface. Note that the Rab9 GTPase binding site indicated at the GCC185 N-terminus is hypothetical; there are Rab GTPase binding sites along the entire length of GCC185, including a dispensible Rab9 site in the bubble region and an AP-1 binding site C-terminal to the bubble (see text).

Very recently, GCC185's N-terminus was also shown to be capable of binding transport vesicles directly, *in vitro* (Cheung et al., 2015). This is consistent with the essential nature of the N-terminal portion for GCC185's roles in both Golgi ribbon maintenance and vesicle capture (Hayes et al., 2009). Using atomic force microscopy, purified GCC185 was shown to occur as a parallel dimer of ~145 nm in length (Figure 3). The N-terminus of most molecules was splayed, and a splayed N-terminus was shown to be most effective in binding transport vesicles *in vitro*.

Given that GCC185 contains an AP-1 binding site just C-terminal to the bubble shown in Figure 3, the vesicle may bind first to the N-terminal “arms” which might then transfer the vesicle to the AP-1 site; alternatively, the vesicle could sit on the AP-1 site and bubble, and be “hugged” by the flexible, N-terminal arms. Moreover, a Rab9 binding site is located near the bubble, but this has been shown to be dispensible for GCC185 function (Brown et al., 2011); perhaps there is additional Rab9-binding site closer to the N-terminus, as indicated in Figure 3. Obviously, additional work is needed to better define the events responsible for vesicle delivery to the TGN.

Another surprise from this study was the finding that GCC185 is significantly shorter than predicted by coiled coil prediction algorithms; moreover, the dimeric coiled coil was unwound about halfway along the length of the protein, creating an internal bubble and making the full length protein appear rather floppy, overall (Figure 3). Deletion of specific, bubble-forming sequences made GCC185 less likely to bend and importantly, incapable of rescuing transport vesicle receipt at the TGN. In addition, the central bubble could be replaced by unstructured, gly-ser repeats in vesicle tethering-rescue experiments, showing that these sequences provide flexibility rather than a partner protein-binding site. These experiments show that GCC185 can bind vesicles at its N-terminus, and also elsewhere along its length, either simultaneously or as part of a multistep process. Vesicle binding by a Y-shaped N-terminus provides avidity for the binding reaction. Importantly, TGN tethers appear to be much more flexible than originally anticipated, capable of collapse onto the Golgi surface to bring vesicles into proximity of the membrane (Cheung et al., 2015; Figure 3).

Wong and Munro (2014) recently used rapamycin-controlled, hetero-dimerization domains to induce relocalization of Golgins to the surface of mitochondria. They found that within only 15 min of rapamycin treatment, membranes containing MPRs and the Vti1a SNARE protein relocalize to mitochondria decorated with any one of three different TGN Golgins: Golgin-245, Golgin-97, and GCC88. Since the Golgins are known to be important for Golgi ribbon maintenance, it was not fully established whether this particular tethering represents trapping of Golgi membranes rather than actual retrograde transport intermediates (especially since Vti1a is a TGN-localized t-SNARE). This is an important distinction because only a tiny proportion of the markers evaluated are present in transport carriers at steady state, yet a large proportion of those marker proteins re-localized to mitochondria in a very short time frame (15 min). If stacks are being relocalized, it would indicate that the TGN tethers studied are most potent in cisternal linking, rather than actual transport intermediate capture. This would easily explain the lack of activity observed for GCC185, a Golgin that is well established to be required for MPR and Shiga toxin retrograde transport in cells (Reddy et al., 2006; Derby et al., 2007; Hayes et al., 2009; Brown et al., 2011) and now shown to bind MPR- and Rab9A-containing vesicles *in vitro* (Cheung et al., 2015).

Wong and Munro (2014) included an anti-CD8 antibody uptake experiment, in conjunction with a CD8-MPR chimera, to be sure that the cargo monitored represented transport from the endocytic pathway; they concluded that Golgin-97, but not GCC185 localizes this construct to mitochondria after 15 min. It takes a CD8-MPR chimeric protein at least 30–45 min to reach the Golgi from the cell surface under normal conditions. In addition, Golgin-97 appears to be a weak tether (if a tether at all) for normal MPR retrograde transport in cells, upon siRNA depletion, as monitored by TGN-specific, tyrosine sulfation of the cation-dependent MPR (Reddy et al., 2006). It will be important to show that Golgin-97 is actually needed for CD8-MPR recycling, which is likely to represent early endosome to Golgi transport rather than late endosome to Golgi transport taken by the native MPR. Nevertheless, Wong and Munro (2014) obtained very beautiful vesicle tethering data for tethers that localize to earlier Golgi compartments.

Are TGN Golgins promiscuous (Wong and Munro, 2014) and do they overlap in function? While this may certainly be true for their Golgi ribbon maintenance activities, TGN Golgins have been shown to differ in their abilities to support distinct retrograde transport pathways. Derby et al. (2007) showed that Shiga toxin, but not TGN38, requires GCC185 for retrograde transport, while TGN38 and MPRs seem to rely on GCC88 (Lieu and Gleeson, 2010). As mentioned above, Reddy et al. (2006) showed that MPR transport relies on GCC185 and much less so, upon Golgin-97. Moreover, depletion of any one of several (but not all), individual Golgins is sufficient to fragment the Golgi into mini-stacks, despite the presence of the other Golgins (Diao et al., 2003; Fridmann-Sirkis et al., 2004; Yoshino et al., 2005; Zolov and Lupashin, 2005; Puthenveedu et al., 2006; Feinstein and Linstedt, 2008). These data suggest that the TGN Golgins are not functionally redundant for transport vesicle tethering.

Golgins contain multiple Rab GTPase binding sites along their lengths (Diao et al., 2003; Beard et al., 2005; Sinka et al., 2008; Hayes et al., 2009; Gillingham et al., 2014). Many of these binding sites are located within regions that are not needed for Golgi localization, implying that Rab:Golgin interactions are involved in vesicle tethering and ribbon maintenance, in addition to Golgi localization. Of the 14 different Rabs that can bind to human GCC185 *in vitro* (Hayes et al., 2009), most are Golgi-localized, but Rab9A is present on inbound transport vesicles (Barbero et al., 2002) and can also bind GCC185. These data imply that vesicle associated Rabs contribute to the vesicle tethering process.

Vesicle-associated Rab proteins can also interact with tethers via linker proteins. The Rab11 effector protein, FIP1/RCP, interacts with Golgin-97 and is required for the retrograde transport of TGN38 (Jing et al., 2010). Golgin-97 binds to the C-terminus of FIP/RCP at a region just upstream of the Rab11-binding domain, suggesting that FIP1/RCP may act like a linker to bring Rab11-containing membranes close to Golgin-97 to mediate the docking of a retrograde carrier.

## Length and shape matter

The length of a tether determines how far it is able to reach out into the cytoplasm. Electron microscopic studies of p115 and its yeast homolog Uso1 showed extended, rod-like structures of their dimeric, carboxy-terminal coiled coil domains (Sapperstein et al., 1995; Yamakawa et al., 1996). The measured lengths of the rod domains of the two proteins are in good accordance with their predicted lengths.

GMAP210 length is important for its function: a mini-GMAP construct containing an intact N-terminal vesicle binding domain, central Rab2 binding domain, and C-terminal GRAB domain cannot rescue Golgi ribbon morphology (Sato et al., 2015). Although apparent tethering activity was not affected when mini-GMAP210 was targeted to mitochondria (Wong and Munro, 2014), the inability to function properly at the Golgi implies that the length of this tether is important for its native function on the Golgi surface. Sato et al. (2015) hypothesize that this includes reaching out through a meshwork of multiple Golgi tethers. It may also be due to the loss of a specific sequence that is important for normal Golgi ribbon maintenance.

GCC185 has been shown to play independent roles in Golgi structure maintenance and transport vesicle tethering (Hayes et al., 2009; Brown et al., 2011). Functional rescue experiments showed that GCC185 does not have to be especially long to support Golgi structure (less than 50% of full length), but a construct of ~60% of its full length, including the first coiled coil domain, is absolutely required for MPR vesicle tethering (Hayes et al., 2009; Brown et al., 2011). Importantly, the full length protein appears to be ~145 nm in length (Cheung et al., 2015), 71% shorter than expected for a protein with such high coiled-coil propensity. Thus, tethering may occur on a shorter distance scale than originally anticipated.

## Golgi ribbon maintenance—clues from unexpected, novel tethers

How do TGN tethers contribute to ribbon maintenance? As mentioned earlier, they contain Golgi-localized, Rab GTPase binding sites along their lengths, and may also interact with additional binding partners that enable them to link regions of the Golgi together. GCC185 N-termini contain Rab and Arl4 binding sites (Hayes et al., 2009; Brown et al., 2011; Lin et al., 2011) and CLASP binding sites for microtubule association (Matsui et al., 2015). Interestingly, GCC185 residues 1331–1573, when removed from the rest of the molecule, render the protein unable to rescue Golgi ribbon morphology. This suggests that the role of this protein in ribbon maintenance must involve a specific, protein:protein interaction that is essential for this aspect of its function and distinct from interactions that drive vesicle tethering. That a region is needed for ribbon maintenance but not for vesicle capture indicates that these processes are also, molecularly distinct.

Support for simple, end-to-end binding reactions as a model to explain tether maintenance of Golgi ribbon structure comes from an unexpected source. KIF1C is an anterograde, kinesin family motor with an N-terminal motor domain. The protein participates in cargo transport from the TGN to the cell surface, and it is also needed to maintain an intact Golgi ribbon structure. Recently, Lee et al. (2015) reported that KIF1C can bind Rab6A at its C-terminus to link this motor to its membrane-bound cargo. More surprising was the observation that KIF1C, but not the related KIF1A protein, can also bind Rab6A via its N-terminal motor domain, and motor domain binding interferes with the ability of the KIF1C motor to bind to microtubules, decreasing the affinity for microtubules by at least 10 fold. KIF1C's ability to bind Rab6A at each end correlated with its ability to maintain an intact Golgi ribbon, even upon nocodazole treatment to disrupt microtubule tracks. In addition, a mutant KIF1C lacking motor activity still had the capacity to maintain Golgi ribbon structure if N- and C-terminal Rab6A binding sites were intact (Lee et al., 2015). These findings suggest that non-Golgin proteins can also function as membrane tethers, and may help transport vesicles hold on to their target membranes to enhance the probability of membrane fusion. The presence of Rab GTPases binding sites at both ends of KIF1C was all that was required to hold the ribbon together. Also, note that Rab6A is on vesicles surrounding and leaving the Golgi. Thus, a molecule of KIF1C anchored on a vesicle via its C-terminal, Rab6A binding domain can be tethered to a Rab6A-containing Golgi domain via the N-terminal motor. As Rab6A becomes less abundant, the motor could instead hop onto a microtubule track and move away from the Golgi complex.

## After binding-what next?

Tethers bind vesicles at a distance, and this interaction is presumed to increase the probability of engagement between vesicle and target SNARE proteins (Pfeffer, 1999). Aside from increasing the chances of target membrane engagement, do tethers do more? The “tentacular meshwork” model (Sinka et al., 2008; Munro, 2011) proposes that tethers act collectively by forming a meshwork surrounding the Golgi. According to this model, Rab binding sites on multiple Golgins entrap vesicles as they undergo multiple rounds of association and dissociation, moving along or between Golgins. Slowing vesicle diffusion will increase the probability of membrane target encounters. This is similar to a “hopping” model in which vesicles hop from one Rab binding site to the next, along a Golgin tether (Hayes et al., 2009; Ramirez and Lowe, 2009), or the “vesicle on a string” concept derived from the fibrous elements detected by electron microscopy connecting *in vitro*-generated COP-I vesicles to Golgi cisternae (Melancon et al., 1987; Weidman et al., 1993; Simon et al., 1996; Orci et al., 1998). Some version of this model is likely to be correct, even if Golgins don't protrude from the surface of the Golgi complex as static, rigid rods. Most Golgin tethers contain breaks between predicted, coiled coil domains that likely represent highly flexible hinge regions (Figure 1). Indeed, the collapsible nature of the purified, GCC185 tether (Figure 3) suggests an alternative structure for the Golgin meshwork that may be shorter and less rigid, but could nevertheless, slow vesicle diffusion.

Finally, some tethering proteins are also able to catalyze SNARE complex assembly. The first indication of such a role came from a study of the early Golgi protein, p115 (Shorter et al., 2002; see also Puthenveedu and Linstedt, 2004). GCC185 binds syntaxin-16 and the interaction can be competed by Rab6A (Ganley et al., 2008); GM130 interacts with Syntaxin-5, and p115 interferes with this interaction (Diao et al., 2008), but the functional consequences of these interactions remain to be determined (Table 1). Much remains to be discovered regarding how long coiled coil tethers might regulate SNARE complex assembly; their SNARE binding capacity may also be equally important to assemble a fusion-competent membrane immediately adjacent to the membrane-anchored, vesicle tether.

## Summary and future perspectives

TGN tethers participate in both Golgi ribbon maintenance and vesicle tethering, and at least for GCC185 and GMAP-210, can do so via distinct and independent domains. These proteins are likely not as long as originally presumed, and structural analysis of each of them will reveal additional clues to their mechanisms of action. Splayed N-termini may represent a more common feature than originally anticipated, providing avidity to tethering reactions. Finally, possible tether collapse onto the Golgi surface will now be an important new mechanism to test more directly, using whatever approaches are available, to provide clues to this interesting, possible new mechanism for vesicle tethering on the Golgi. How do tethers release their cargoes? Why don't cytosolic pools of tethers interfere with the tethering process? Why does the Golgi ribbon fragment so readily? Clearly there remains a great deal yet to discover.

## Author contributions

All authors listed, have made substantial, direct and intellectual contribution to the work, and approved it for publication.

## Funding

This research was funded by a grant from the NIH (DK37332).

### Conflict of interest statement

The authors declare that the research was conducted in the absence of any commercial or financial relationships that could be construed as a potential conflict of interest.
